# Case report: ALK D1225N missense mutation in lung adenocarcinoma responds to tyrosine kinase inhibitors

**DOI:** 10.3389/fphar.2023.1190447

**Published:** 2023-07-17

**Authors:** Jianxin Chen, Junhui Wang

**Affiliations:** ^1^ Department of Medical Oncology, The Quzhou Affiliated Hospital of Wenzhou Medical University, Quzhou People’s Hospital, Quzhou, Zhejiang, China; ^2^ Department of Radiation Oncology, The Quzhou Affiliated Hospital of Wenzhou Medical University, Quzhou People’s Hospital, Quzhou, Zhejiang, China

**Keywords:** ALK gene, missense mutations, tyrosine kinase inhibitors, lung adenocarcinoma, non-small-cell lung cancer

## Abstract

*ALK* gene missense mutations are conventionally considered non-driver mutations without pathological significance, and therefore, there is a lack of effective target drugs against them. The standard treatment option for patients with *ALK* missense mutations is chemotherapy with or without antiangiogenic agents, which usually results in unsatisfactory outcomes. Herein, we present the case of a patient with metastatic lung adenocarcinoma harboring the only missense mutation in *ALK* D1225N responding to two ALK-tyrosine kinase inhibitors (TKIs), namely, crizotinib and ensartinib. Our case highlights that non-small cell lung cancer (NSCLC) patients harboring the D1225N mutation may benefit from ALK-TKIs, and therefore, ALK-TKIs should be considered candidates for further line treatment.

## 1 Introduction

tAnaplastic lymphoma kinase (ALK), a transmembrane tyrosine kinase encoded by the *ALK* gene localized on chromosome 2, is a member of the superfamily of insulin receptors (Fukui et al., 2022). Several cell signaling pathways, including the signal transducer and activator of transcription 3 (STAT3) and AKT/PI3K pathways, are regulated by *ALK* expression to maintain biological functions, including cell proliferation, cycling, and survival (Bai et al., 2000; Chiarle et al., 2005; Kasprzycka et al., 2006). To date, *ALK* alterations, including fusion, rearrangement, and missense mutations, have been associated with the occurrence, rapid progression, and metastasis of carcinomas, including neuroblastoma, rhabdomyosarcoma, and non-small-cell lung cancer (NSCLC) (van Gaal et al., 2012; Bresler et al., 2014; Schneider et al., 2023). Approximately 3%–8% of patients with NSCLC harbor *ALK* rearrangement mutations, which are conventionally considered the main driver mutations among *ALK* alterations (Kapoor et al., 2023; Rossi et al., 2023). Accordingly, several ALK tyrosine kinase inhibitors (TKIs), including crizotinib, ceritinib, alectinib, lorlatinib, brigatinib, and ensartinib, have been approved for the treatment of NSCLC patients with *ALK* fusion mutations because of their promising efficacy in well-designed trials (Fukui et al., 2022). However, *ALK* missense mutations are conventionally considered non-driver mutations, and therefore, there is a lack of effective target agents against them. Herein, we report, to the best of our knowledge, for the first time, a patient with metastatic lung adenocarcinoma harboring a missense mutation in *ALK*, namely, the D1225N mutation, showing response to ALK-TKIs crizotinib and ensartinib. Owing to the rarity and thought-provoking nature of the case, we hope that this case presentation offers insights to physicians for detailed investigations in basic experimentation.

## 2 Case presentation

**FIGURE 1:**
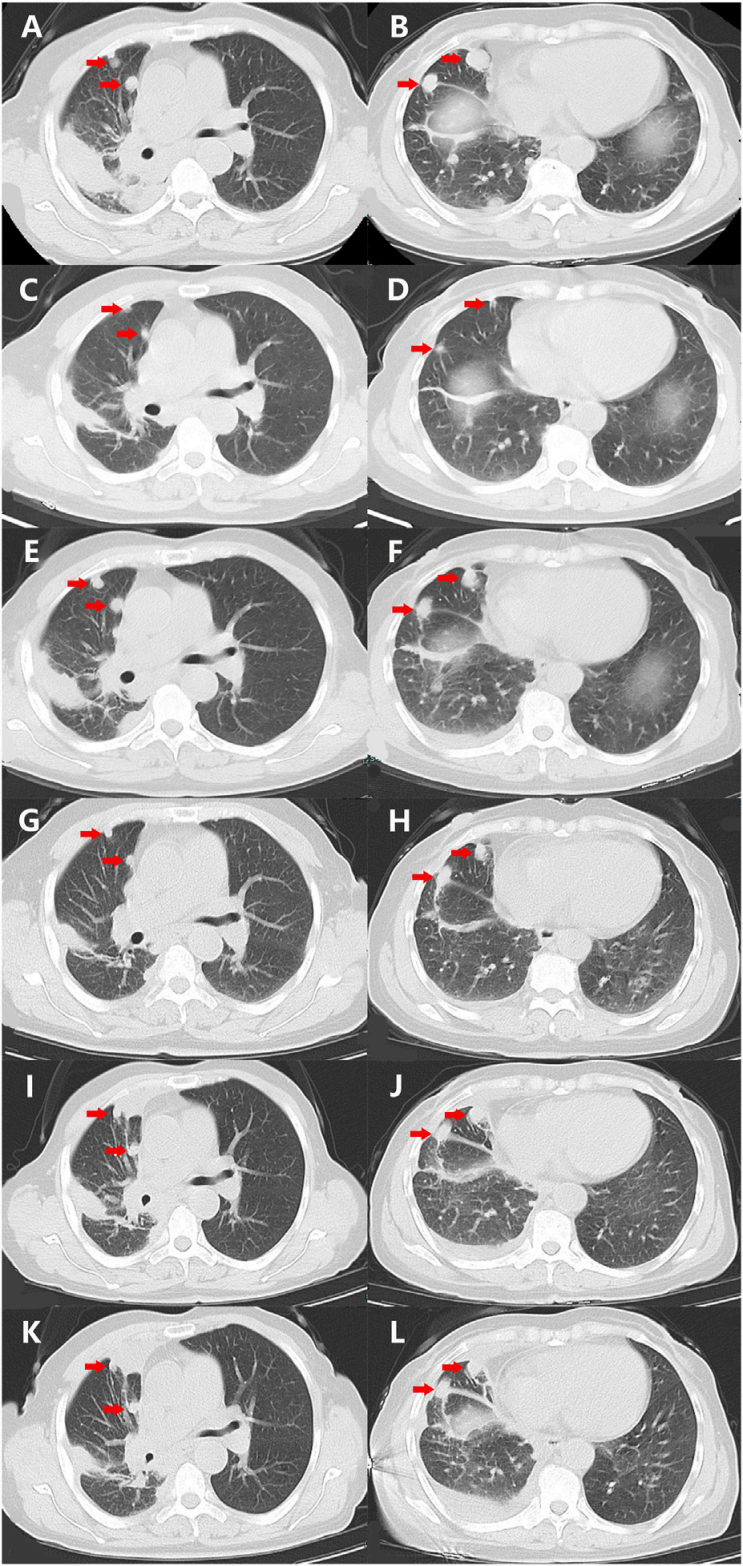
Variations of the primary and metastatic lesions in lung detected by chest CT scans during the treatment (red arrowheads). **(A,B)** Chest CT scans showed the primary and metastatic lesions (red arrowheads) in the lungs on 20 November 2021. **(C,D)** Chest CT scans showed the primary and metastatic lesions (red arrowheads) in the lungs on 5 January 2022. **(E,F)** Chest CT scans showed the primary and metastatic lesions (red arrowheads) in the lungs on 15 March 2022. **(G,H)** Chest CT scans showed the primary and metastatic lesions (red arrowheads) in the lungs on 12 April 2022. **(I,J)** Chest CT scans showed the primary and metastatic lesions (red arrowheads) in the lungs on 29 June 2022. **(K,L)** Chest CT scans showed the primary and metastatic lesions (red arrowheads) in the lungs on 25 July 2022. Abbreviations: CT, computerized tomography.

**FIGURE 2:**
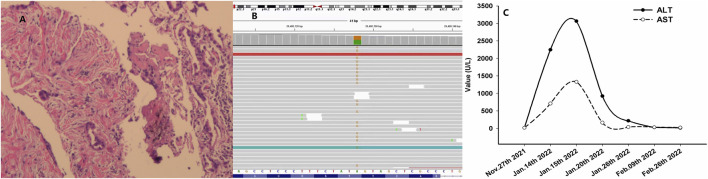
Histological findings with hematoxylin and eosin stain, NGS, and variation of ALT and AST. **(A)** Histological findings with biopsy from percutaneous puncture of the lesion in the right lung; **(B)** NGS results showing missense mutations of ALK D1225N (column in yellow color for the specific missense mutation as ‘G’); and **(C)** variation of ALT and AST during the treatment. Abbreviations: NGS, next-generation sequencing; ALT, alanine transaminase; AST, aspartate transaminase; and ALK, anaplastic lymphoma kinase.

A 68-year-old Chinese woman was admitted to Quzhou People′s Hospital on 19 November 2021, complaining of a cough lasting approximately a month. She had no history of smoking, alcohol intake, or any other medical condition or hereditary disease. A chest computed tomography (CT) was performed on 20 November 2021, and multiple space-occupying lesions in the right lung were revealed (Figures 1A, B), accompanied by enlargement of the lymph nodes in the right hilum and mediastinum. In addition, no other distant suspicious lesions were detected on abdominal CT or brain MRI. Subsequently, a percutaneous puncture biopsy was performed, the results of which revealed invasive lung adenocarcinoma (Figure 2A) based on the following immunohistochemistry findings: TTF-1 (positive), NapsinA (positive), P40 (negative), P63 (negative), CK7 (positive), CDX-2 (negative), Syn (negative), CgA (negative), Ki-67 10% (positive), and programmed cell death ligand-1 (PD-L1) (negative). Based on those findings, the patient was clinically diagnosed with invasive lung adenocarcinoma with metastatic lesions in different lobes, along with metastasis to the mediastinal lymph nodes, which was staged as IVB (cT4N2M1) according to the criteria of the American Joint Committee on Cancer (AJCC) 8th edition. In addition, the patient’s DNA (sample from tumor tissue, tumor cellularity 20%, detection depth in target area 970.75, and target area coverage 99.33%) was subjected to tumor driver gene profile testing via next-generation sequencing (NGS, Biomed Diagnostics, Shanghai), which revealed an anaplastic lymphoma kinase (*ALK*) p. D1225N alteration caused by an exon 24 c.G3673A missense mutation (Figure 2B). The referred transcript adopted for ALK analysis in the present case was NM_004304.

## 3 Diagnostic assessment

As no targetable mutations were detected, palliative systemic therapy with chemotherapy combined with an anti-PD-1 inhibitor was suggested. However, the patient refused a regimen of cytotoxic drugs and was discharged on 27 November 2021. After 7 weeks, the patient was admitted to our outpatient clinic with abdominal distension for 3 days on 14 January 2022. A liver function test with the serum sample showed extremely elevated alanine transaminase (ALT, 2246.8 U/L, normal range 4.0–48.0 U/L, Figure 2C) as well as elevated aspartate transaminase (AST, 710.3 U/L, normal range 4.0–42.0 U/L, Figure 2C). She was immediately hospitalized because of serious liver injury. She acknowledged that she had taken crizotinib at a dose of 250 mg twice daily, as suggested by another patient. The patient denied consuming any other medicines. In addition, she also acknowledged that repeated chest CT on 5 January 2022 revealed substantial regression (partial response, 85%) of the primary tumor as well as metastatic tumors in the lung (Figures 1C, D). Nevertheless, we still suggested discontinuation of crizotinib because of severe liver function injury, potentially caused by crizotinib. With comprehensive and effective treatment, the elevated ALT and AST levels decreased to the normal range (Figure 2C). A chest CT was conducted again after liver function recovery on 15 March 2022, and the results revealed enlargement of the tumors again (Figures 1E, F). Owing to the efficacy of crizotinib observed in the front-line treatment, subsequent therapy with ensartinib, a second-generation ALK-TKI, was administered at a dose of 225 mg once per day after sufficient discussion with the patient. After a month of ensartinib administration, efficacy assessment with repeated chest CT revealed partial response (PR) (Figures 1G, H), and it continues (Figures 1I, J). Ensartinib treatment was terminated due to progressive disease on 25 July 2022 (Figures 1K, L). No adverse events were observed during ensartinib treatment. Thereafter, the patient was administered systemic therapy with pemetrexed, carboplatin, bevacizumab, paclitaxel, and anti-PD-1 inhibitors. Currently, the patient is receiving nab-paclitaxel combined with penpulimab as a second-line treatment. Her progression-free survival (PFS) time on ALK-TKIs is 8 months, with an overall survival of 16 months and it continues.

## 4 Discussion

Herein, we report a case of metastatic lung adenocarcinoma in a patient harboring the D1225N missense mutation in *ALK* that responded to ALK-TKIs, namely, crizotinib and ensartinib. Although the PFS by ALK-TKIs lasted only 8 months, which was much less than that seen with *ALK* fusion mutations, we observed regression of primary and metastatic tumors during treatment. To the best of our knowledge, this case is the first report of the response of a patient harboring the missense D1225N mutation in *ALK* to ALK-TKIs in clinical practice. This case suggests that the D1225N mutation might be sensitive to TKIs and may be a candidate for salvage treatment in select patients. Because of the rarity and thought-provoking nature of the case, we hope that the presentation of the case will offer insights to physicians and encourage detailed investigations and basic experimentation.

*ALK* plays a significant physiological role in brain development and can be mutated oncogenically in several carcinomas, including NSCLC and anaplastic large cell lymphoma (ALCL) (Holla et al., 2017). The most prevalent and targetable ALK mutations are chromosomal rearrangements that result in gene fusion, as observed in ALCL and NSCLC (Holla et al., 2017). In addition, several missense mutations in *ALK* have been suggested to significantly affect tumorigenesis in NSCLC (Wang et al., 2011). Missense mutations, including H694R, E1384K, V597A, G881D, S413N, and Y1239H, have been identified in preclinical studies to lead to tumor development through the activation of signal molecules, including STAT3, AKT, and ERK (Wang et al., 2011). In these models, ALK promotes the activation of downstream signaling pathways as well as other crucial pathways of malignant phenotypes, such as uncontrolled cellular proliferation and division. However, to date, no effective agent targeting these missense mutations has been developed. In the present case, we observed tumor regression after two ALK-TKI treatments, suggesting that the *ALK* D1225N mutation is sensitive to ALK-targeted therapy. Moreover, because of the limited duration of response (DoR) of such missense mutations, further investigation to understand its potential underlying mechanism of action is needed.

*ALK* missense mutations occasionally occur in NSCLC patients harboring fusion alterations who are resistant to ALK-TKIs. Common secondary mutations include F1174L, F1174C, L1196M, I1171T, G1202R, S1206Y, G1269S, and G1269A (Choi et al., 2010; Sasaki et al., 2010; Heuckmann et al., 2011; Doebele et al., 2012). Although several *ALK* missense mutations have been discovered, most ALK-TKI-resistant tumor cells continue to depend on ALK signaling and are sensitive to more potent, structurally distinct, and further-generation ALK-TKIs (Sakamoto et al., 2011; Crino et al., 2016). However, in the present study, the specific mutation of *ALK* D1225N was not reported as a secondary mutation after TKI treatment, even though the mutation site was located in the structural domain of the *ALK* gene, which suggests that the mutation may be primarily sensitive to TKIs. Moreover, in addition to NSCLC, the *ALK* mutation D1225N has been reported in other carcinomas, including rhabdomyosarcoma and pediatric cancers, in which it has been considered a driver alteration (van Gaal et al., 2012; Takita, 2017). However, the biological cause of the activation of the *ALK* D1225N mutation in rhabdomyosarcoma remains controversial; consequently, there are no potential targeted drugs against D1225N (Yoshida et al., 2013). In addition, there is no literature reporting missense mutations in NSCLC, according to the search results of Genecards (www.genecards.org).

In the present study, crizotinib treatment led to hepatotoxicity with severe ALT and AST elevation and was classified as grade 4 (life-threatening consequences) by NCI Common Terminology Criteria for adverse events version 5.0 (NCI CTC AE). We speculated crizotinib administration as the main cause of hepatic failure. The patient recovered with effective liver protection treatment. The switch to ensartinib proved to be safe after recovery from hepatic failure.

Further identification of the effectiveness of ALK-TKIs against specific missense mutations in *ALK* and their mechanism of action should be investigated. In addition, we have undertaken a project to verify this clinical phenomenon by mimicking the D1225N point mutation using gene editing in a basic experiment.

In brief, we present a case of metastatic lung adenocarcinoma characterized by the *ALK* D1225N missense mutation, which has conventionally been considered a non-driver mutation, and demonstrate its partial response to ALK-TKIs. Our case highlights that patients with NSCLC harboring the *ALK* D1225N missense mutation may benefit from ALK-TKIs and should be considered candidates for further treatment.

## Data Availability

The original contributions presented in the study are included in the article/Supplementary Materials, further inquiries can be directed to the corresponding author.
